# A study of the dosimetric impact of daily setup variations measured with cone‐beam CT on three‐dimensional conformal radiotherapy for early‐stage breast cancer delivered in the prone position

**DOI:** 10.1002/acm2.13080

**Published:** 2020-10-30

**Authors:** Annie Xiao, Jessica Jutzy, Greg Hubert, Meghan Edens, Maxine Washington, Yasmin Hasan, Steven J. Chmura, Hania A. Al‐Hallaq

**Affiliations:** ^1^ Pritzker School of Medicine The University of Chicago Chicago IL USA; ^2^ Department of Radiation and Cellular Oncology The University of Chicago Chicago IL USA; ^3^ Department of Radiation Oncology The University of Minnesota Minneapolis MN USA; ^4^ Department of Radiation Oncology Winship Cancer Institute of Emory University Atlanta GA USA

**Keywords:** breast cancer, cone‐beam CT, prone positioning, setup margins, whole‐breast radiotherapy

## Abstract

**Purpose:**

To evaluate the dosimetric impact of daily positioning variations measured with cone‐beam computed tomography (CBCT) on whole‐breast radiotherapy patients treated in the prone position.

**Methods:**

Daily CBCT was prospectively acquired for 30 consecutive patients positioned prone. Treatment for early‐stage (≤II) breast cancer was prescribed with standard dose (50 Gy/25 fractions) or hypofractionation (42.56 Gy/16 fractions) for 13 and 17 patients, respectively. Systematic and random errors were calculated from the translational CBCT shifts and used to determine population‐based setup margins. Mean translations (±one standard deviation) for each patient were used to simulate the dosimetric impact on targets (PTV_eval and lumpectomy cavity), heart, and lung. Paired Student’s t tests at α = 0.01 were used to compare dose metrics after correction for multiple testing (*P* < 0.002). Significant correlation coefficients were used to identify associations (*P* < 0.01).

**Results:**

Of 597 total fractions, 20 ± 13% required patient rotation. Mean translations were 0.29 ± 0.27 cm, 0.41 ± 0.34 cm, and 0.48 ± 0.33 cm in the anterior–posterior, superior–inferior, and lateral directions leading to calculated setup margins of 0.63, 0.88, and 1.10 cm, respectively. Average three‐dimensional (3D) shifts correlated with the maximum distance of breast tissue from the sternum (r = 0.62) but not with body‐mass index. Simulated shifts showed significant, but minor, changes in dose metrics for PTV_eval, lung, and heart. For left‐sided treatments (n = 18), mean heart dose increased from 109 ± 75 cGy to 148 ± 115 cGy. Shifts from the original plan caused PTV_eval hotspots (V105%) to increase by 5.2% ± 3.8%, which correlated with the total MU of wedged fields (r = 0.59). No significant change in V95% to the cavity was found.

**Conclusions:**

Large translational variations that occur when positioning prone breast patients had small but significant dosimetric effects on 3DCRT plans. Daily CBCT may still be necessary to correct for rotational variations that occur in 20% of treatments. To maintain planned dose metrics, unintended beam shifts toward the heart and the contribution of wedged fields should be minimized.

## 1 INTRODUCTION

Breast‐conserving surgery (BCS) is currently considered the standard surgical treatment for early‐stage breast cancer over mastectomy due to equivalent survival and improved cosmesis.^1^, ^2^ Given the excellent local disease control of BCS when paired with adjuvant radiation therapy, recent research has focused on improving normal tissue toxicities.

Whole‐breast radiotherapy (WBRT) after BCS is historically delivered to the patient in the supine position. In patients with large breast separation, however, dose inhomogeneity can result in worse fibrosis.^3^ Additionally, pendulous breast anatomy could exhibit larger daily setup variability due to arbitrary shifts of breast position by gravity.^4^ Attempts to mitigate this problem with angled breast boards can result in increased breast to skin contact over the inframammary folds^5^, ^6^ and higher heart and lung tissue doses.^4^, ^6^

To better address these problems, prone positioning has been evaluated for treatments during which large breast size or pendulous breast tissue is of concern. This approach has improved dose homogeneity leading to excellent cosmetic outcomes,^7^, ^8^, ^9^ while also providing dosimetric advantages by reducing intrafraction setup variation from respiratory motion,^4^, ^10^ minimizing lung volumes receiving 10 and 20 Gy, and increasing displacement of breast tissue from the chest wall, heart, and contralateral breast.^11^, ^12^ Prone positioning is particularly powerful for reducing the heart dose to as low as possible,^5^, ^6^ which is especially important in light of the study by Darby et al that demonstrated a no‐threshold relationship of dose to chronic heart toxicities in breast cancer patients.^13^

Despite these demonstrated dosimetric advantages, interfraction variability of breast tissue and the necessity of daily setup image guidance remain uncertain. Prone WBRT is additionally challenging because the anatomical site is not directly visualized and the patient lies on deformable tissue (i.e., the contralateral breast).^14^ Studies evaluating prone breast setup for WBRT using cone‐beam CT (CBCT) have demonstrated daily setup variations necessitating a clinical target volume (CTV) to planning target volume (PTV) margin of 1.0–2.2 cm.^10^, ^14^, ^15^ While margins are not typically utilized for WBRT plans, they provide a consistent way to compare both systematic and random setup uncertainties among various institutions. Previous studies focused on comparison of prone to supine positioning^10^, ^11^, ^14^, ^16^ or on the impact of setup error on partial breast irradiation.^4^, ^17^ More recent studies have estimated the dosimetric effects of positioning variations as quantified from planar images but not from CBCT.^18^, ^19^ We hypothesized that daily CBCT is necessary in these patients to achieve accurate positioning, particularly to correct for rotations and deformations that we had regularly observed, in order to ensure accurate dose delivery during treatment. To our knowledge, this study is the first to evaluate the dosimetric impact of daily positioning variations measured with CBCT on WBRT. Here, we report on the impact that both setup margins and patient characteristics have on the dosimetry of three‐dimensional conformal radiotherapy (3DCRT) plans and on the role of CBCT for prone WBRT.

## 2 MATERIALS AND METHODS

Data from 30 consenting and unselected patients who received CBCT‐guided prone WBRT at The University of Chicago Medicine from May 2015 through March 2017 were prospectively collected (IRB# 16352A) in consecutive order. Surgical management included lumpectomy alone for patients with ductal carcinoma in situ (DCIS) or lumpectomy with sentinel lymph node evaluation for those with invasive breast cancer. Prone WBRT was limited to patients with node‐negative disease requiring breast‐only treatment. Hypofractionation was utilized for patients who did not require chemotherapy, lumpectomy cavity boost, or who were >50 yr as it has been shown to provide acceptable acute toxicity rates at our institution.^8^, ^9^

### 2.A Radiotherapy planning and delivery

**Fig. 1:**
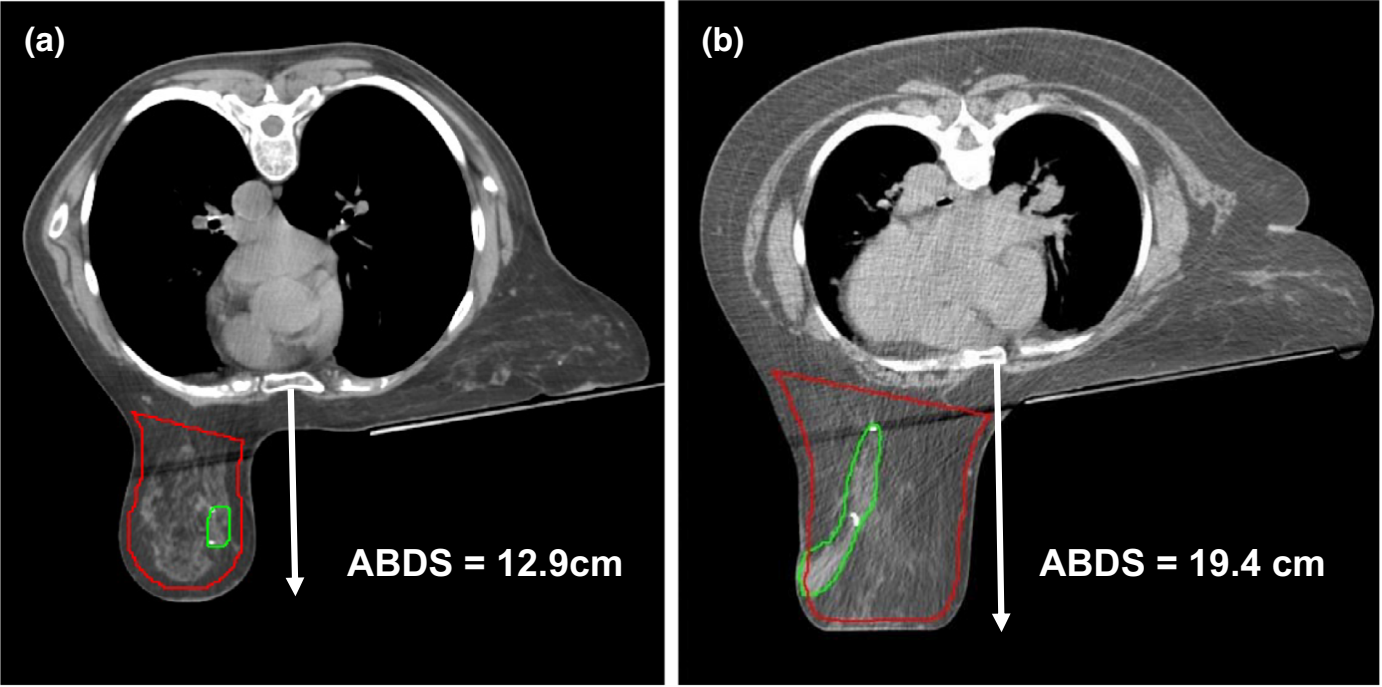
Anterior breast distance from the sternum as a measure of breast pendulousness for two patients, with PTV_eval (red contour) and lumpectomy cavity (green contour) overlaid. Breast tissue contacted the Styrofoam base for the patient on the right.

Patients were simulated using a CDR Systems prone breast board with indexable handles (CDR Systems Inc., Calgary, AB, Canada) with the addition of upper and lower alpha cradles. While patients lie flat on the prone device, the board underneath the contralateral breast was either flat (n = 5) or angled (n = 25). Simulations were performed on a Brilliance BigBore CT scanner (Philips Healthcare, Andover, MA) using 3‐mm slice thickness. Three‐dimensional conformal radiotherapy plans were created using high‐energy photon beams (6–18 MV), physical or computerized wedges, and/or large segments for optimization of dose homogeneity and target coverage as described previously.^8^ A structure to evaluate target dose (i.e., PTV_eval) was generated to encompass all breast tissue within the tangential beams but excluding the tissue in the buildup region (i.e., ~6 mm from the skin). At our institution, this structure is generated automatically using the 70% isodose line calculated from the unmodified tangential beams and edited by physicians if necessary. The lumpectomy cavity was contoured to include the seroma (n = 29) plus any visible surgical clips when present (n = 24). Figure 1 shows target volumes from two representative patients. The anterior breast distance from the sternum (ABDS), depicted in Figs. 1(a) and 1(b), was measured in each plan as a surrogate for the pendulousness of the breast tissue. Dose using tissue density correction was calculated for each plan intended for treatment using Pinnacle v9.8 (Philips Healthcare, Andover, MA).

Patients were treated on a Varian linear accelerator (2100EX) equipped with an OBI console (Varian Medical Systems, Palo Alto, CA). Marks placed on the back, including a three‐point localization (i.e., on the spine and lateral aspects of the torso) in a plane centered on the breast, guided initial patient positioning. The three‐point localization was tattooed at the time of simulation and marks to localize the treatment isocenter were added and reapplied daily as needed. CBCT was used for daily alignment and repeated following any adjustments of the patient’s posture. The CBCT was registered manually to the planning CT to account for translations with a focus on matching the chestwall and any surgical clips in the breast while also ensuring that the contralateral breast was sufficiently away from the field edge. If significant postural rotations or tissue deformations in either the ipsilateral or contralateral breast were identified, the patient was repositioned on the prone board and a second CBCT was acquired to ensure that these had been corrected and to implement any remaining translational shifts. Thus, the second CBCT inherently contained a priori knowledge about the patient’s position. For example, if the therapy team determined that the patient should be shifted laterally relative to the indexed prone board in order to keep the contralateral breast away from the field edge, they would have done so while also correcting any other postural discrepancies. As a result, the final recorded lateral shift following the second CBCT would have been smaller.

### 2.B Margin calculations

Margins to account for setup uncertainty in each dimension were calculated using the van Herk recipe: 2.5 × Σ + 0.7 × σ, where Σ and σ are standard deviations for systematic and random errors, respectively. While the calculated margin does not account for rotations, it ensures coverage by at least 95% of the prescribed dose to the CTV for 90% of patients in whom translations are not corrected daily via image guidance.^20^ First, translational shifts from CBCT in the anterior/posterior (A/P), superior/inferior (S/I), and right/left (R/L) dimensions were tabulated and the mean shift (M) and standard deviation (STD) were calculated for each patient. Note that the mean shift M includes the direction of the shift depending upon the sign associated with the shift magnitude. Then, total setup error was calculated from the mean of M across patients, Σ was calculated from the standard deviation of M across patients, and σ was calculated from the root mean square of STD across patients. This methodology has been applied to breast patients treated in different fractionation regimens by others.^14^

#### 2.B.1 Dose calculation for plans with simulated shifts

To simulate the dosimetric effects of uncorrected systematic and random setup errors, the original treatment plan was recalculated using the mean setup errors (i.e., M ± STD) for each patient in every dimension and each direction. Doses were tabulated and maximum deviations from the intended treatment plan were compared to institutional constraints for the following dose metrics: V95% (volume receiving 95% dose) for PTV_eval and lumpectomy cavity targets, V105% (volume receiving 105% dose) for PTV_eval, mean heart dose, and V20 Gy (volume receiving 20 Gy) for ipsilateral lung.

#### 2.B.2 Statistical analysis

Linear regression using R statistical package (http://www.R‐project.org) was used to identify significant correlations (*P* < 0.01) between: (a) average 3D shifts and patient characteristics (age, BMI, ABDS), and (b) dosimetric changes from simulated shifts and shift magnitudes (mean, standard deviation), the use of wedges (total MU of wedged fields), or patient characteristics (age, BMI, ABDS). Analysis of the correlation coefficients was repeated for the subset of patients treated with 16 and 25 fractions. Multivariate analysis was used to determine the importance of each parameter on the overall correlation if more than 1 parameter was found to correlate significantly. To compare setup errors across patients in subgroups, the random errors were compared using a two‐tailed *t* test and systematic errors were compared using an *F* test (*P* < 0.01) as reported by Feng et al.^21^ Paired Student’s *t* tests at α = 0.01 were used to compare dose metrics across plans after Bonferroni correction for multiple testing resulting in a *P* < 0.002 (i.e., *P* < 0.01/5).

## 3 RESULTS

### 3.A Patient characteristics

Patient characteristics are shown in Table 1. Of 30 patients, 25 were overweight or obese. Mean heart dose constraints per RTOG 1304^22^ (i.e., <400 cGy) were met for all patients and ranged from 19 to 323 cGy.

**Table 1:** Patient and treatment characteristics.

Characteristic	
Number of patients	30
Median age (yr) [range]	62 [46–88]
Body mass index (kg/m^2^)	
Median [range]	29 [20.5–45.6]
18.5–25 (normal)	5
>25–30 (overweight)	11
>30 (obese)	14
Stage	
0	4
1	18
2	8
Dose and fractionation	
Standard (50 Gy, 25 fractions)	13
Hypofractionated (42.56 Gy, 16 fractions)	17
Average of mean heart dose (cGy) [range]	84.2 [19–323]
Average of ipsilateral lung V20 Gy (%) [range]	0.33 [0–3.5]

### 3.B Daily inter‐fractional setup error

A total of 597 CBCT images were acquired for 30 patients. Patients required postural correction and a repeat CBCT scan after the initial CBCT in 19.6%± 13% of treatment fractions. In the remainder of 479 fractions, the terminal CBCT scan was used to implement any residual translations ≥ 1 mm in any direction; in 32 of the 479 fractions no translations were deemed necessary.

Table 2 shows the means of absolute shifts and total setup errors, as well as standard deviations of systematic and random errors, calculated from the translations indicated by the terminal CBCT (i.e., after all postural corrections had been implemented) in 597 treatment fractions. The total setup error combines the positive and negative shifts, resulting in smaller total error values than absolute shifts. After excluding the 19.6% of fractions with a priori information (i.e., from the initial CBCT which indicated that postural correction was necessary), the random and systematic errors calculated from the remaining 479 fractions did not differ significantly. The random and systematic errors were also not significantly different for the subset of patients treated with 16 or 25 fractions compared to those from all 597 fractions.

**Table 2:** Absolute translational shifts, total errors, standard deviations of systematic and random errors, and setup margins in three dimensions for 597 treatments and comparison of margins for subset analyses.

	A/P	S/I	L/R
Absolute shifts (±SD, cm)	0.29 (±0.27)	0.41 (±0.34)	0.48 (±0.33)
% shifts within 5 mm	83.4	68.7	61.3
% shifts within 7 mm	92.0	80.9	75.4
% shifts within 9 mm	99.8	98.2	96.5
Total setup error (cm)	0.05	−0.20	−0.09
SD of systematic error (cm)	0.15	0.23	0.29
SD of random error (cm)	0.37	0.46	0.52
**Setup margin (cm)**	**0.63**	**0.88**	**1.10**
*Setup margin for 479 fractions (cm)*	*0.62* ^a^	*0.92* ^a^	*1.12* ^a^
*Setup margin for 16 fractions (cm)*	*0.64* ^a^	*0.98* ^a^	*1.15* ^a^
*Setup margin for 25 fractions (cm)*	*0.60* ^a^	*0.75* ^a^	*1.05* ^a^

^a^ Systematic and random errors used to calculate these margins did not differ significantly (*P* < 0.01) from those used to calculate the setup margins from 597 treatments.

### 3.C Margins for prone whole‐breast radiotherapy

The systematic and random errors from CBCT measurements were used to estimate a setup margin using the van Herk’s population‐based formula. To ensure 95% target coverage for 90% of patients when positioned to skin marks alone without image‐guided radiotherapy (IGRT) per van Herk’s methodology, an anisotropic margin of 0.63 cm (A/P), 0.88 cm (S/I), and 1.10 cm (R/L) would be required. These setup margins represent the lower threshold for margins required to account for setup errors because they do not account for rotational errors per van Herk’s methodology.^20^ When margins were recalculated for patients treated with different fractionation regimens, small changes were observed for patients treated in 16 fractions (i.e., increases of 0.01–0.10 cm) and 25 fractions (i.e., decreases of 0.03–0.13 cm) as shown in Table 2. Margins recalculated from the 479 treatment fractions in which only a single CBCT was acquired were likewise comparable: 0.62 cm (A/P), 0.92 cm (S/I), and 1.12 cm (R/L).

### 3.D Effect of patient characteristics on interfractional setup error

We evaluated the associations between patient age, BMI, the pendulousness of breast tissue via ABDS, and the average 3D shifts. Only significant correlations are reported here (*P* < 0.01). Data from three patients were excluded from the correlation coefficient analysis as their breast tissue was distorted due to contact with the Styrofoam base as shown in Fig. 1(b). These three patients had ABDS >16 cm, which potentially would have been larger in the absence of the treatment table. The remaining values of ABDS ranged from 5.6 to 17.2 cm.

**Fig. 2:**
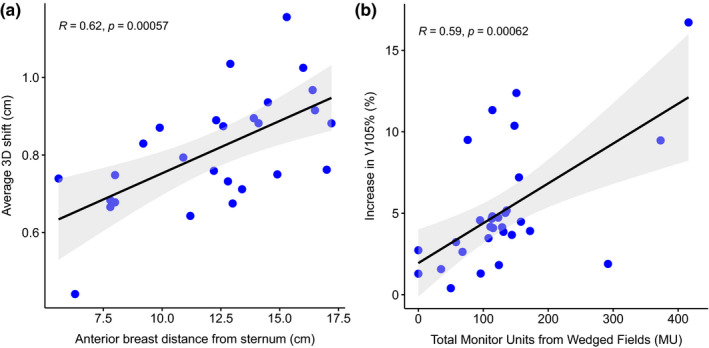
(a) Three‐dimensional average shift correlates significantly (r = 0.62) with the anterior breast distance from sternum after excluding data points for patients whose breast was in contact with a Styrofoam base. (b) Increases in V105% to the PTV_eval in simulated plans correlate significantly (r = 0.59) with the total monitor units from wedged fields. In both plots, the linear fit is shown in a black line while the 95% confidence interval is shown in light gray shading.

Significant correlations including their 95% confidence intervals are summarized in Table 3. Correlations were additionally evaluated for the following subsets of data: (a) patients treated with differing fractionation regimens, and (b) CBCT shift data without a priori information (n = 479). We found that patients with a larger ABDS required larger average 3D shifts than those with less pendulous breasts, with a correlation coefficient of 0.62 [Fig. 2(a)]. Although average 3D shifts correlated with ABDS, they did not correlate with BMI despite the fact that ABDS and BMI were moderately correlated with each other (r = 0.55). This was confirmed with multivariate analysis of the effects of both BMI and ABDS on average 3D shifts. Correlation coefficients increased for the subset of patients treated in 16 fractions but were not significant for patients treated in 25 fractions. This could be due to the fact that ABDS correlated with BMI for the patients treated in 16 fractions (r = 0.63) but not in 25 fractions. The correlations were also higher when recalculated for treatment fractions (n = 479) in which a single CBCT was acquired (i.e., no a priori information). The strongest correlation occurred when average 3D shifts were analyzed for the 497 fractions with a single CBCT and for patients treated in 16 fractions (r = 0.78).

**Table 3:** Correlation coefficients and their 95% confidence intervals for various patient characteristics including body‐mass index (BMI), anterior breast distance from sternum (ABDS), and average three‐dimensional (3D) shifts calculated from daily cone‐beam computed tomography (CBCT) with (n = 597) and without a priori information (n = 479). Analyses are repeated for the subset of patients treated in 16 fractions. Patients with breasts that contacted the treatment table (n = 3) were removed from the analysis. Only significant correlation coefficients (*P* < 0.01) are listed.

	BMI	BMI	ABDS	ABDS
	All (n = 27)	16 fractions (n = 16)	All (n = 27)	16 fractions (n = 16)
ABDS	0.55 [0.21–0.77]	0.63 [0.19–0.86]	NA	NA
Average 3D shift (n = 597)	NS	NS	0.62 [0.31–0.81]	0.73 [0.37–0.90]
Average 3D shift (n = 479)	0.51 [0.16–0.75]	NS	0.68 [0.40–0.84]	0.78 [0.46–0.92]

	Wedged MU	Wedged MU
	All (n = 30)	16 fraction (n = 17)
V105% to PTV_eval	0.59 [0.29–0.78]	0.77 [0.46–0.91]

NA, not applicable; NS, not significant.

### 3.E Dosimetric impact of plans using simulated shifts

**Fig. 3:**
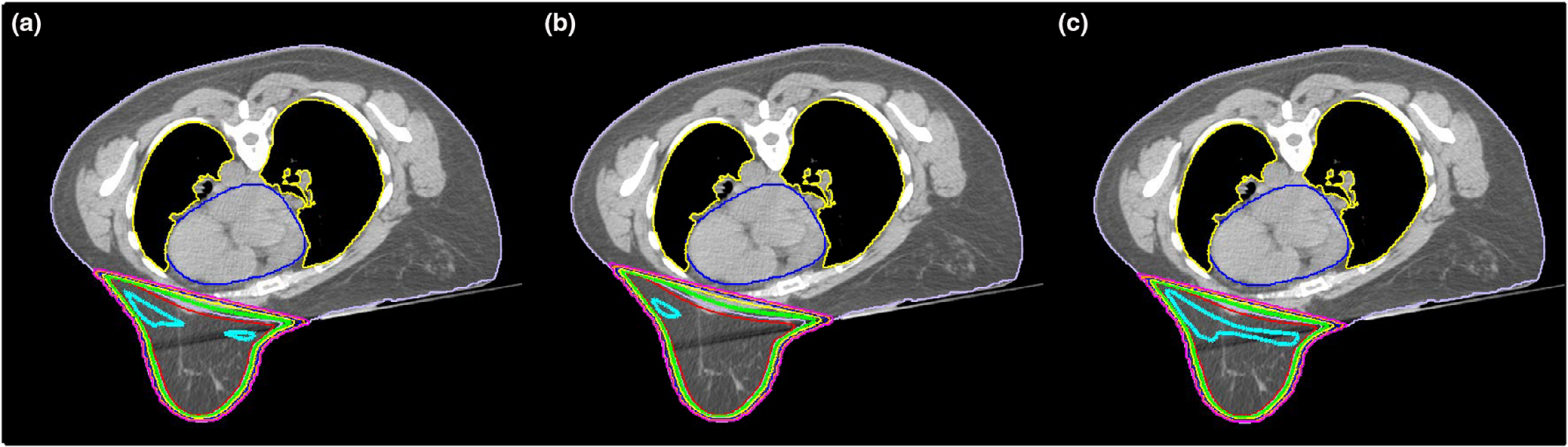
Isodose lines of 95% (green) and 105% (cyan) overlaid on an axial computed tomography image demonstrate the (a) intended dose and dose for simulated shifts of the patient (b) by 0.48 cm anteriorly toward the beam isocenter (c) and by 0.35 cm posteriorly away from the beam isocenter (M = −0.06 cm, STD = 0.42 cm). Dose to the heart increases from 0.86 to 1.1 Gy when the patient is shifted anteriorly (b) while the volume of V105% increases from 11.2% to 16.4% when the patient is shifted posteriorly (c).

The magnitude of shifts used to simulate the dosimetric impact of setup variations ranged from 0.13 to 0.82 cm across all dimensions with an average of 0.43 ± 0.13 cm across patients. Table 4 reports dose metrics from intended and simulated plans reflecting maximal deviations from institutional constraints. No significant change in dose coverage to the lumpectomy cavity was found. Simulated plans showed significant, but small, changes in dose metrics for PTV_eval, heart, and lung. Most notably, the volume of the PTV_eval receiving ≥ 95% of the prescription dose (i.e., V95%) decreased by 1.6 ± 1.1%, the volume of ipsilateral lung receiving ≥ 20 Gy (i.e., V20 Gy) increased by 0.4 ± 0.6%, and mean heart dose for left‐sided treatments increased from 109 ± 75 cGy to 148 ± 115 cGy. Simulated shifts of the patient anteriorly toward the beam isocenter as shown in Fig. 3, which reduced the distance between the beam edge and the heart, were the cause of the increased heart dose for the majority of plans (16 of 18) and for ≥1% increases in lung V20 in four plans. The 105% hotspots (i.e., V105%) to the PTV_eval increased by 5.2% ± 3.8%, and correlated (r = 0.59) with the total monitor units (MU) of wedged fields as shown in Fig. 2(b). This correlation increased to 0.77 for the patients treated in 16 fractions. Despite statistically significant changes in dose, simulated plans exceeded institutional dose constraints in only one plan (V105% for PTV_eval) in which all fields were wedged.

**Table 4:** Dose metrics for intended and simulated plans using patient‐specific mean shifts with one standard deviation (M ± STD).

Variable	OAR	Intended plan	Simulated plan max deviation	Institutional constraint	Number of simulated plans violating constraint	Institutional “variation acceptable” constraint	Number of simulated plans violating secondary constraint	*P* value
Mean dose (cGy)	Heart^b^	109 ± 75	148 ± 115	<400	1	<500	0	<0.001^a^
V20Gy (%)	Lung	0.4 ± 0.8	0.8 ± 1.3	<15%	0	<20%	0	<0.001^a^
V95% (%)	PTV_eval	98.8 ± 1.2	97.2 ± 1.9	>95%	4	>90%	0	<0.001^a^
V95% (%)	Lumpectomy Cavity	98.0 ± 5.7	97.9 ± 5.9	N/A^c^	N/A	>95%	3^d^	<0.005
V105% (%)	PTV_eval	12.9 ± 5.1	18.0 ± 7.1	<20%	13	<30%	1	<0.001^a^

^a^ Significant difference between intended and simulated plans.

^b^ Reported only for left‐sided cancer (n = 18).

^c^ Institutional constraint is V100% > 95%.

^d^ Same three plans did not meet criterion in original plan; lumpectomy cavity from one patient could not be visualized on CT scan and was not delineated.

## 4 DISCUSSION

Prone positioning has been successfully used for WBRT following breast‐conserving surgery, but formal guidelines for use of IGRT for setup verification have yet to be outlined.^12^ Prior WBRT studies of prone positioning using daily CBCT have demonstrated interfraction variability requiring a margin of 1.0–2.2 cm.^10^, ^14^, ^15^ Along a similar vein, we found that a setup margin of ~1.0 cm in the S/I and R/L dimensions is necessary for adequate target coverage if alignment were made solely to skin marks independent of the fractionation regimen. However, the setup margin in the A/P dimension was smaller than 1 cm in our study (0.63 cm). Our positioning workflow also includes alpha cradle immobilization to index patients to the prone board and treatment table, enabling fixation of couch position in three dimensions. Fixing the couch position is expected to reduce setup errors, similarly to a study by Petillion et al.^23^ which demonstrated a decrease in the A/P positioning error when the couch vertical position was fixed for WBRT in the supine position. In a comparison of supine and prone positioning, Kirby et al.^10^ corroborated that setup errors for prone positioning could potentially be reduced by several factors: optimization of the prone board design, placement of tattoos, increasing staff experience, and/or verification by CBCT. Mulliez et al noted that studies of margins for prone positioning report discordance in the dimension requiring the largest margin and postulated that this could be related to the prone board design.^14^ Other studies using different prone devices found that the largest setup margin is required in the A/P,^16^ R/L,^14^, ^15^ or the S/I dimension.^10^ In our study, in which patients lie flat on a prone board and daily CBCT was used, we found that the A/P margin is smaller than in other dimensions.

To elucidate the role of CBCT for ensuring proper dose delivery in prone WBRT, we performed dose calculations simulated using the patient‐specific mean shifts M including the standard deviation. The dose metrics of these simulated shifts demonstrated minor but significant deviations from the intended dose, with only one plan exceeding institutional dose constraints. This is perhaps expected given that WBRT delivered using large, predominantly open tangential fields is fairly robust to setup variability.^24^ Even intensity modulation has been shown to mainly affects the hotspots and not the OAR doses for prone WBRT.^25^ It should also be noted that the simulated shifts were estimated from CBCT shifts which were smaller in the A/P direction than others have found. Because A/P shifts would potentially have the largest impact on doses to the heart and lungs, it is possible that these dosimetric changes would have been larger if daily CBCT were not used. In fact, several studies have shown that the use of daily IGRT for left‐sided breast cancer patients was necessary to ensure the lowest heart doses during treatment.^18^, ^19^ Although minor dosimetric degradation of plans was found in our simulations, the goal at our institution is to minimize cardiac doses as much as possible in order to reduce potential cardiac toxicities.^1^, ^13^ By using CBCT daily, we can accomplish this goal while also ensuring that the breast shape is reproduced for treatment in order to limit dose inhomogeneity which has been associated with acute radiation dermatitis.^26^

Although daily CBCT had a small but significant effect on the planned dose, there are other reasons for its use, such as for quality control^18^ or for an in‐house investigation of setup variability^27^ as in the current study. Similarly to the rationale presented by Mulliez et al.,^14^ we use CBCT to correct the patient’s roll and ensure that the contralateral breast tissue is outside of the treatment field. In our study, postural adjustments requiring rotations were necessary in approximately 20% of treated fractions (19.6 ± 13%) and cannot be accounted for using PTV margins.^20^ Moreover, the use of PTV margins is not typical for 3DCRT in breast radiotherapy due to the increased dose that would be delivered to OARs although such margin calculations would be applicable for prone partial breast irradiation.^17^ Instead, daily CBCT allowed us to correct the patient’s posture and provided our therapy team with valuable information about patient positioning.

The use of daily imaging for patients who are at risk for less reproducible setup, as in the case of prone treatments, is in concordance with the recent ASTRO guidelines for WBRT.^28^ More recently, studies of prone breast patients that have used planar kV imaging for IGRT have concluded that daily IGRT is necessary to ensure adequate target coverage^18^ while minimizing the heart dose.^18^, ^19^ Our study goes one step further by using CBCT to identify rotations and tissue deformation that required postural corrections of patients in 20% of the treatment fractions. While a quarter of the patients required infrequent postural corrections (<10%), frequent postural corrections (≥25%) were necessary in one third of patients. Since we could not identify any parameter to predict which patients would require these postural corrections and do not believe that planar imaging can adequately identify soft tissue deformations in the breast shape particularly for the contralateral breast (on which the patient lays) as alluded to by others,^14^, ^19^ we would advocate for the use of daily CBCT rather than kV imaging. A direct comparison between the two methods, which have been shown to yield comparable imaging doses,^29^ could not be accomplished at our institution as we only acquire CBCT for treatments. However, Feng et al compared the margins calculated from CBCT acquired following kV‐guided patient positioning of supine postmastectomy patients and found that residual errors ranging from 4 to 8 mm persisted despite the use of kV.^21^ This discrepancy between kV and CBCT would be expected to be larger when including deformable breast tissue and thus highlights the utility of 3D imaging in localizing the breast in the prone position.^14^

Dose calculations of simulated shifts additionally revealed that improvements to plan robustness could potentially be achieved by minimizing unintended A/P shifts of the patient to limit heart dose and by reducing the total MUs of wedged fields, which correlated with increases in V105%. Increased hotspot volumes have been associated with increased acute skin toxicities,^30^ particularly for hypofractionated treatments,^8^, ^9^ which can in turn lead to poor cosmesis.^3^ Of note, the simulated plan that did not meet institutional constraints was developed for a patient with less pendulous breast tissue, with ABDS of 5.6 cm, which was below the median ABDS of 13.0 cm. Unique to this patient was that all treatment fields were wedged. Because wedges are not customized to patient anatomy, plans with wedges suffered greater dosimetric degradation when calculated following simulated shifts leading to a larger associated increase in V105% to PTV_eval. Thus, limiting the use of wedges for future plans may improve the plan robustness.

Prone positioning is more often used for patients who have pendulous or sizeable breasts that lend to increased interfraction variability in the supine position due to the effects of gravity. Of previous studies, only Mulliez et al^14^ described a relationship between BMI and setup error in the A/P and R/L dimensions. We evaluated the patient metrics that result in increased interfraction setup variation, including patient BMI and ABDS as a proxy for breast pendulousness. Correlation analysis showed a moderate positive correlation between ABDS and interfraction variability as quantified by average 3D shifts, but no significant correlation between BMI and average 3D shifts. In fact, multivariate analysis indicated that ABDS was significantly predictive of average 3D shifts but BMI was not. These data suggest that skin marks on the torso and/or bony alignment using kV imaging may not be sufficient surrogates for pendulous breast tissue suffering the effects of gravity. Alternatively, ABDS may be a better surrogate for the expected magnitude of shifts because it is a more direct measure of the pendulousness of the breast. The correlation between ABDS and average 3D shifts was more tightly coupled in treatment fractions without a priori knowledge about the patient’s position (n = 479). Moreover, patients treated in 16 fractions had larger correlations between average 3D shifts and pendulous breast tissue (i.e., ABDS) which may have been due to the fact that the correlation between ABDS and BMI reached significance in this subset of patients. However, due to the small number of patients receiving hypofractionated treatment these results may not necessarily generalize to a larger dataset or other institutions.

There are some limitations in our study. Shifts simulated by displacing the CT simulation data do not account for residual deformations that could occur in breast shape on a daily basis. This effect is expected to be small in our study compared to others who have made this assumption without the added benefit of 3D imaging^18^, ^19^ since CBCT led our therapy team to correct the patient’s posture and subsequently any gross deformations in breast shape prior to treatment. Another limitation is that the CBCT scans acquired for positioning could not be used to perform dose calculations because of significant artifacts that stem from truncation of patient anatomy, much of which extend outside of the CBCT field‐of‐view. These artifacts are similar to those shown in the study by Jozsef et al.^17^ Also, the use of a mean shift to simulate the day‐to‐day variations could have overestimated the true dosimetric changes. To temper this potential overestimate, one STD was added to the mean shift rather than two STD. Finally, the simulated dosimetric changes in V95% and V105% to PTV_eval may not necessarily reflect changes in dose to breast tissue. The PTV_eval structure represents breast tissue within the tangential fields and has been used by RTOG protocols (e.g., RTOG1005) to limit the target from extending outside of the treatment fields or into the thorax/lungs. As a result, this structure served as a surrogate for the target in our clinic and did provide insight into the dosimetric impact of shifts detected by our study. As shown in Fig. 1, PTV_eval predominantly encompasses breast tissue.

## 5 CONCLUSION

Our work demonstrates that in 3DCRT WBRT plans without intensity modulation, setup variations caused small but significant dosimetric changes although further gains could be achieved by minimizing anterior shifts of the patient toward the beam to limit increases to the heart dose and by reducing the total monitor units of wedged fields to limit increases in target hotspots. While these results appear to downplay the importance of daily CBCT for reproducing the planned dose to targets, forgoing CBCT may have occasionally delivered higher than planned dose to the heart and would have risked treating patients with uncorrected postural rotations/deformations in 20 ± 13% of fractions. Based on the results of our study, daily CBCT is recommended for prone positioning for 3DCRT plans.

## CONFLICT OF INTEREST

No conflict exists.
